# Body typing of children and adolescents using 3D-body scanning

**DOI:** 10.1371/journal.pone.0186881

**Published:** 2017-10-20

**Authors:** Henry Loeffler-Wirth, Mandy Vogel, Toralf Kirsten, Fabian Glock, Tanja Poulain, Antje Körner, Markus Loeffler, Wieland Kiess, Hans Binder

**Affiliations:** 1 Interdisciplinary Centre for Bioinformatics, Leipzig University, Härtelstraße 16–18, Leipzig, Germany; 2 LIFE, Leipzig Research Center for Civilization Diseases; Leipzig University, Philipp-Rosenthal-Straße 27, Leipzig, Germany; 3 Hospital for Children and Adolescents, Centre for Pediatric Research; Leipzig University, Liebigstraße 20a, Leipzig, Germany; 4 Institute for Medical Informatics, Statistics and Epidemiology, Leipzig University, Härtelstraße 16–18, Leipzig, Germany; Vanderbilt University, UNITED STATES

## Abstract

Three-dimensional (3D-) body scanning of children and adolescents allows the detailed study of physiological development in terms of anthropometrical alterations which potentially provide early onset markers for obesity. Here, we present a systematic analysis of body scanning data of 2,700 urban children and adolescents in the age range between 5 and 18 years with the special aim to stratify the participants into distinct body shape types and to describe their change upon development. In a first step, we extracted a set of eight representative meta-measures from the data. Each of them collects a related group of anthropometrical features and changes specifically upon aging. In a second step we defined seven body types by clustering the meta-measures of all participants. These body types describe the body shapes in terms of three weight (lower, normal and overweight) and three age (young, medium and older) categories. For younger children (age of 5–10 years) we found a common ‘early childhood body shape’ which splits into three weight-dependent types for older children, with one or two years delay for boys. Our study shows that the concept of body types provides a reliable option for the anthropometric characterization of developing and aging populations.

## Introduction

Anthropometry is important for understanding the development of children and adolescents. It allows for detailed evaluation of diversity of body shapes and their variations in the context of aging and disorders. Measures such as the body mass index (BMI) [1], the waist circumference [2], the waist-to-height-ratio [3] and the neck circumference [4] are used to evaluate the nutritional status, and to detect obesity and the risk of related secondary diseases. Overweight, obesity and their comorbidities are a widespread problem in children and adolescents with a need for measures that differentiate between normal physiological and pathological development [5].

Three-dimensional (3D-) whole body scanning is a relatively new technique to gather anthropometric data in medicine, although the scanning technology itself has been available for some time. Today most of the scanning systems are laser-based and generate a three-dimensional mesh of the scanned object using optical triangulation [6]. The scanning of whole persons is widespread in commercial applications like apparel design and ergonomics [7,8]. Most applications for medical purpose, however, focus on single parts of the human body only, e.g. for cosmetic and reconstructive surgery [9,10]. The most important advantage of body scanning technology here remains unused, which is the opportunity to gather dozens of individual body measures at once with high accuracy [11,12] and within only a few seconds of time. On the other hand, 3D-body scanning produces large sets of data, which need new algorithms and approaches for 3D-shape analysis including normalization and dimension reduction [13].

3D-body scanning was systematically applied in the Leipzig Research Center for Civilization Diseases (LIFE) to adult persons and to children. The later ones were assessed in the LIFE Child study which is one of the largest longitudinal studies with an extensive phenotyping of urban children and adolescents in Germany [14]. It has recently completed the 11,000^th^ examination of more than 3,500 children from Leipzig, Germany. The general objective of LIFE Child is to evaluate the development of children and to detect early onset markers of major civilization diseases. Participants undergo an extensive assessment program including anthropometry, structured interviews, questionnaires, physical examinations and biospecimen collection [15].

Main aim of this publication is to pursue a novel analysis strategy based on the stratification of children into a set of well-defined body types, and their description in terms of age-dependent changes. We expect that this approach will provide a complementary description scheme for anthropometric data with possible impact for disease-related associations, e.g., in the context of obesity. We here present an analysis approach for a large set of multidimensional 3D-laser scanning body measures of children and adolescents in the age range between 5 and 18 years. We systematically analyzed body measures collected in LIFE Child by 3D-body scanning of 2,735 children and adolescents to define novel anthropometric phenotypes. This data constitutes, to the best of our knowledge, one of the largest sets of such data presently available. For this, we utilized and extend the methodical framework for body scanner data of adult persons developed recently [13]. We describe clusters of related body measures and how to utilize them for stratification of participants into distinct body types.

## Material and methods

### Ethics approval and consent to participate

The LIFE Child study has been registered with the trial number NCT02550236 and was approved by the Ethics Committee of the University of Leipzig (Reg. No. 264-10-19042010). As a prerequisite to enrolment, written informed consent was obtained from all participants or their parents. All procedures performed in studies involving human participants were in accordance with the ethical standards of the institutional research committee and with the 1964 Helsinki declaration and its later amendments [16]. The data privacy and safety concept of the study was approved by the responsible data protection officer.

### 3D-body scanning

In this study we analyzed anthropometric 3D-body scanner data collected in the LIFE Child study between 2011 and 2016. A comprehensive description of the study design is given in [15]. In brief, in total about three thousand healthy children and adolescents were recruited in the area of Leipzig and assessed in a multi-method approach in one-year follow-ups over ten years. The assessments performed in this study varied for different age groups ranging from pregnancy to the age of 18 years, and comprised inter alia interviews, questionnaires, classical anthropometry, biological sampling, and 3D-body scanning. The latter was performed by a ‘Vitus Smart XXL’ 3D-laser scanner (Human Solutions GmbH, Kaiserslautern, Germany) which provides a model of the body surface of each participant. Body measures were extracted from this model using AnthroScan 2.9.9 software (Human Solutions GmbH). Scanner and software comply with the ISO 20685 international standard.

Data were provided by the LIFE research data base after thorough curation. It contained 46 measures including 18 (linear) lengths and distances, 25 (curved) girths, weight, and the aggregated characteristics ‘body mass index’ (BMI [1]), and ‘waist circumference to height ratio’ (WHtR [3]).

### Preprocessing

We obtained body scanner data of 2,735 children and adolescents (1,379 boys and 1,356 girls). The body measures of each participant were preprocessed as described in [13]. In short, each measure of a participant was first divided by the body height. Then, each measure was Z-normalized, i.e. centralized with respect to its mean value averaged over all participants, and divided by its standard deviation. Z-normalization makes the different measures comparable by providing a common scale in units of the standard deviation of each measure in the sample.

### SOM clustering of body measures

We recently developed an analysis workflow based on self-organizing maps (SOM) to evaluate body scanner data [13], which was applied to the preprocessed LIFE Child data: In the first step, SOM machine learning was used for unsupervised clustering of the 46 body measures, delivering eight clusters called ‘meta-measures’. These meta-measures collect between 1 and 15 single measures. Their values were calculated as mean values averaged over the respective single measures in each of the clusters. The set of meta-measures anthropometrically characterizes the whole body of each participant. We Z-normalized the eight meta-measures of each participant to remove redundancies in the sets of meta-measures due to additive ‘offsets’. These normalized body meta-measures were subsequently used as input data for clustering participants into body types as described below.

### Consensus clustering of participants

Consensus clustering and dynamic dendrogram cutting was applied to cluster participants with similar body measures. For input data we utilized the eight Z-normalized meta-measures characterizing all 2,735 participants. Consensus clustering is a bootstrapping method to evaluate cluster stability using sub-sets of randomly sampled participants [17]. It provided a quadratic consensus matrix with the frequency of common cluster memberships for all pairwise combinations of participants in a series of 100 resampling runs as elements. This consensus matrix was then clustered using hierarchical clustering. Finally, separate body types were defined by dynamic dendrogram cutting [18]. For details see S1 Text.

### Availability of data

The minimal data set consists of height- and z-normalized body scanner data of all participants together with the assignment of sex, age and body type. It is provided as S1 Table and will enable interested researchers to perform their own clustering and class discovery, and to reproduce findings and conclusions presented in this manuscript. Raw data underlie consortial data safety restrictions, but these data can be requested from the LIFE Consortium (www.life.unileipzig.de/en/).

## Results and discussion

### Meta-measures collect body measures with similar profiles

We applied SOM machine learning to reduce dimensionality of the 46 individual body scanner measures in our data (see S1 Text for details). In brief, the method aggregated the body scanner data into clusters of correlated measures [13]. We obtained eight clusters of anthropometric measures termed meta-measures, each containing between 1 and 15 individual body measures. The complete list of meta-measures and of assigned body measures is given as S2 Table.

**Fig 1:**
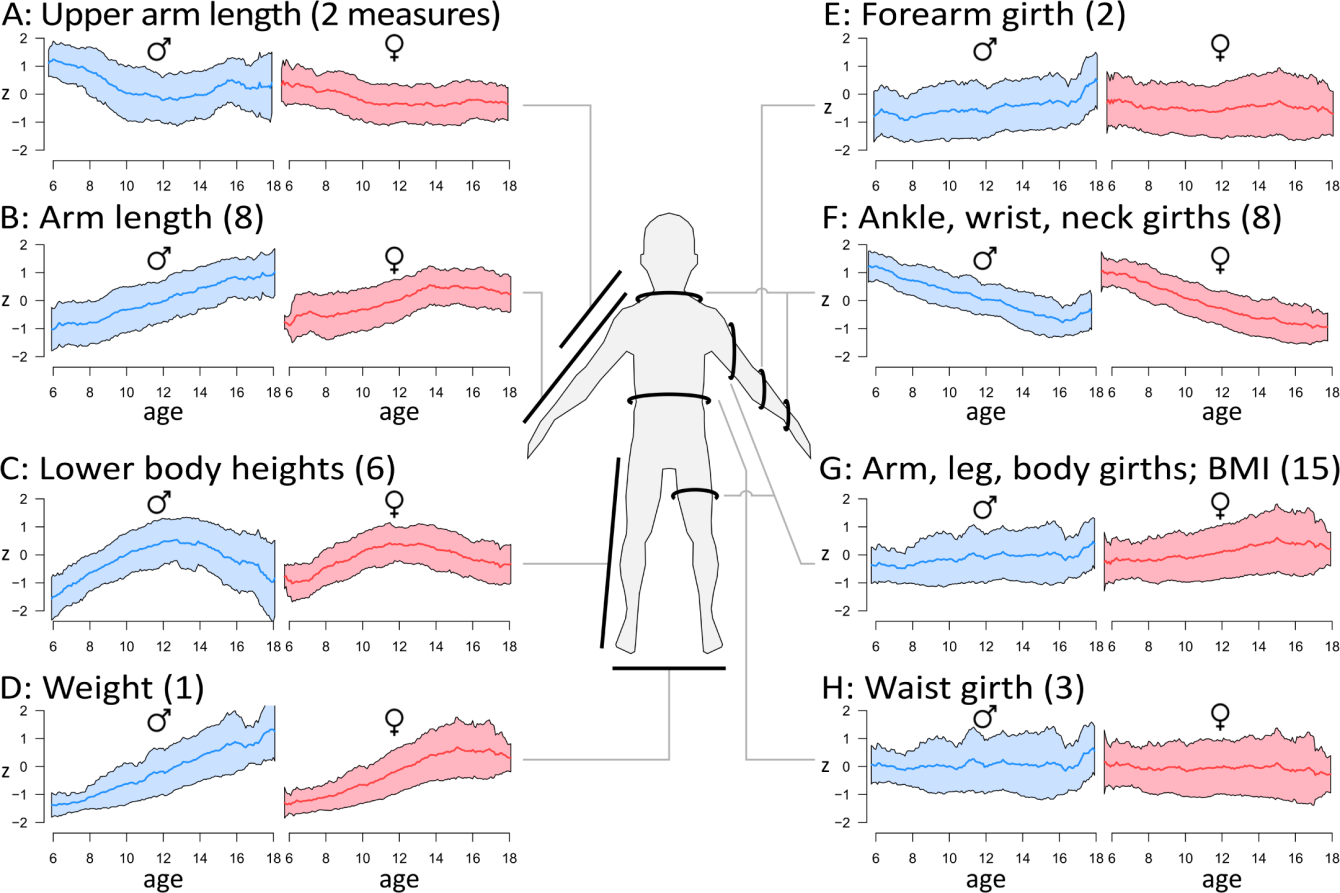
Body meta-measures as a function of age. The profiles reflect different types, e.g. monotonous growths and decays, profiles with a maximum or minimum at intermediate ages, and also almost invariant measures. The body measures were normalized with respect to body height and standard deviation providing a Z-scale.

Fig 1 shows the meta-measure values stratified according to participants’ sex and age. They are given in standard deviation units after Z-normalization during preprocessing, and they represent scaled body measures in relation to body height. The profiles can be divided into meta-measures with increasing (see ‘B’ and ‘D’ in Fig 1) and decreasing (‘F’) values upon development, but also age independent meta-measures (‘H’ and, to a lesser extent, ‘E’ and ‘G’) with almost similar courses for boys and girls are observed. Meta-measures with bimodal behavior can be seen as indicators of pubertal changes in body shape: Upper arm length (‘A’) increases slower than body height up to an age of about 10, leading to the decrease of the meta-measure value due to height normalization. Afterwards the opposite effect is observed for boys, whereas upper arm measures in girls grow proportionally with body height in agreement with previous results [19,20]. Lower body heights (‘C’, including knee and hip heights, and inseam lengths) are representative for leg length. They increase over-proportionally compared to the body height until an age of about 12 years giving rise to the increase of the relative measure used here. Then, the relation of growth-velocities reverses and the relative measure for lower body heights starts to decrease. Hence, the maximum of the curve reflects completion of strong leg growth in favor of growth of other body parts.

Taken together, clustering of the 46 body measures into 8 meta-measures reduces dimensionality and provides age-dependent profiles which characterize development on an aggregated level of information.

### Distinct body types associate with developmental stages and overweight characteristics

**Fig 2:**
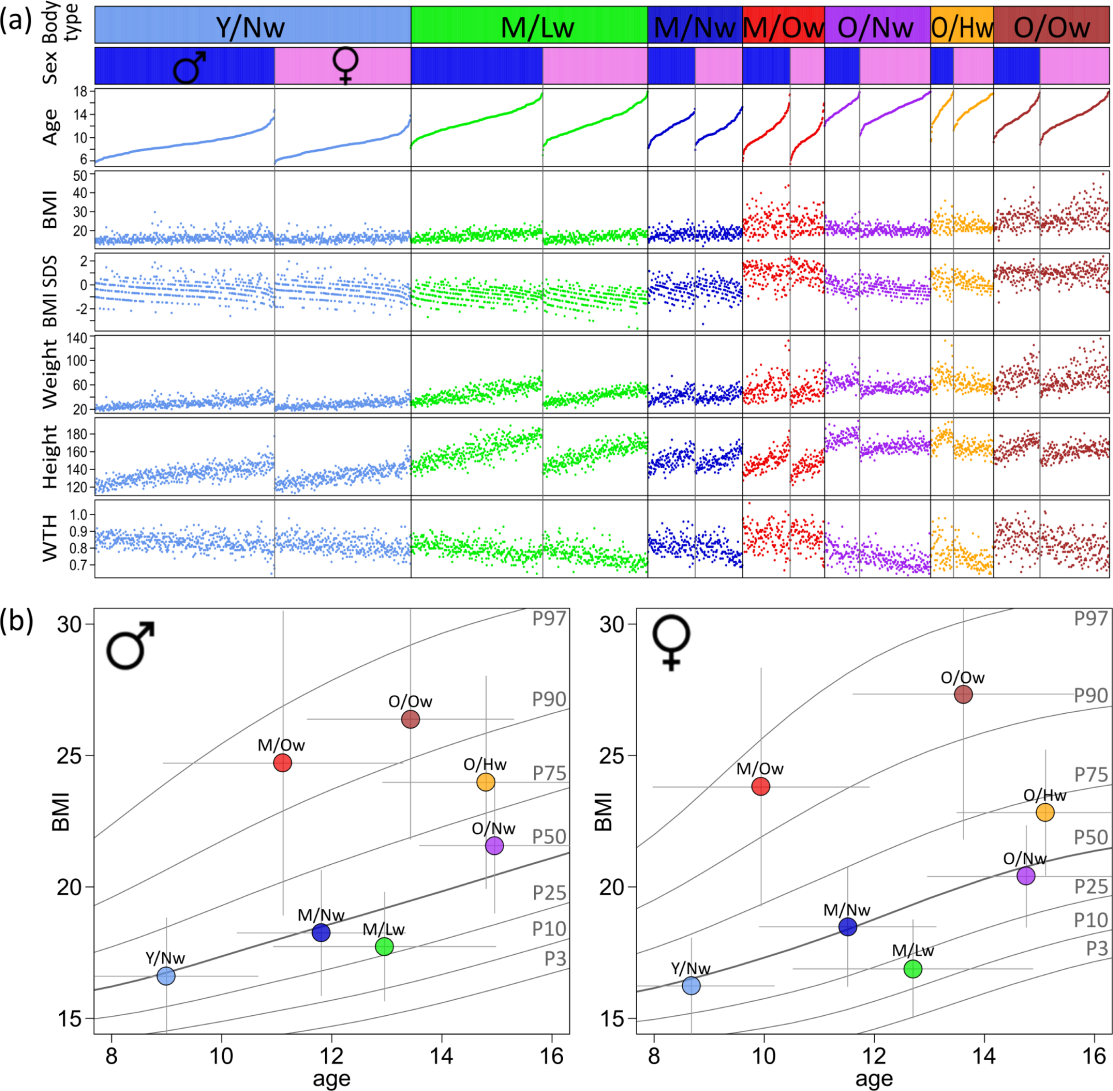
Characterization of the body types as seen by classical anthropometry. (a) Measures and indices with individual resolution. Participants are ordered according to body type, sex and age. Each dot represents one participant. (b) Mapping mean age and BMI of body types into percentile curves of boys and girls, respectively. The bars indicate standard deviation within each body type.

The eight meta-measures reduce the dimensionality of the original 46 body measures. We used this data for diversity analysis of all 2,735 participants by applying sample clustering. In total we extracted a set of seven distinct body types. Their basic characteristics are given in Table 1 and in Fig 2A. We found that the body types systematically differ in mean age and BMI characteristics. For a rough classification, the body types were therefore termed by combining information on mean age (‘Y’,’M’ and ‘O’ denote young, medium and older age) and BMI (‘Lw’, ‘Nw’, ‘Hw’ and ‘Ow’ denote lower, normal, high and overweight participants, see Table 1 for nomenclature). We plotted mean age and BMI of each of the body types–separately for boys and girls–into percentile curves characterizing German children and adolescents as provided by the Robert Koch Institute [21] (see Fig 2B). The curves confirm that the body types cover a range of different stages of physiological development for different BMI categories. Note that the localization of the male and female body types in the plot is very similar, with a slight trend to lower BMI for girls. Changes of the BMI characteristics with increasing age are discussed below.

**Table 1:** Description of the body types. Additional information and stratification according to male and female participants can be found in S1 Text.

Body type	Age & weight categories	# Individuals	Age (y) ^1^	BMI ^1^	BMI SDS ^1^
**Y/Nw**	**Y**ounger participants, **N**ormal **w**eight	852	8.9 ± 1.6	16.4 ± 2.1	-0.39 ± 0.71
**M/Lw**	**M**edium age, **L**ower **w**eight	640	12.9 ± 2.1	17.3 ± 2.0	-0.88 ± 0.63
**M/Nw**	**M**edium age, **N**ormal **w**eight	255	11.7 ± 1.6	18.4 ± 2.3	-0.33 ± 0.68
**M/Ow**	**M**edium age, **O**ver**w**eight / obese	220	10.6 ± 2.2	24.3 ± 5.3	0.98 ± 0.77
**O/Nw**	**O**lder participants, **N**ormal **w**eight	287	14.8 ± 1.7	20.8 ± 2.2	-0.21 ± 0.55
**O/Hw**	**O**lder participants, **H**igh **w**eight	169	15.0 ± 1.7	23.2 ± 3.1	0.26 ± 0.59
**O/Ow**	**O**lder participants, **O**ver**w**eight / obese	312	13.6 ± 2.0	27.0 ± 5.2	0.95 ± 0.59

1: average value ± standard deviation.

The ‘Y/Nw’-body type (‘young/normal weight’) collects most of the participants. It refers to youngest children showing an infantine body shape (852 participants, with an average age of 9 years) in agreement with [19]. Note that there is virtually no other body type collecting participants in this age range meaning that the body shape is relatively uniform up to an age of approximately 10 years. Only a few participants in the youngest age range are assigned to ‘M/Ow’ (‘medium age/overweight’), showing that they already acquired overweight body shape in this early age (see also below).

Two body types are located at the 95-percentile (P95) lines, namely ‘M/Ow’ and ‘O/Ow’. They cover the broad age range from 11 to 15 years and divide obese participants into two differing body shapes. Please note that the age ranges of these two body types overlap for boys, but virtually split into two separated ranges for girls (see standard deviation bars in Fig 2B). This finding suggests the parallel development of two distinct body shapes of overweight boys, whereas overweight girls seem to undergo a body type change at about 12 years of age. A similar development can also be observed in the normal weight ‘Nw’-types, where transitions from ‘Y/Nw’ to ‘M/Nw’ and from ‘M/Nw’ to ‘O/Nw’ are evident at about 10 and 13 years, respectively [19].

In summary, the seven body types relate to different age ranges and to different BMI classes. They constitute a classification which involves information on the whole body shape rather than utilizing a subset of only very few measures as realized, e.g., in traditional BMI and waist-to-hip indices.

### Body types reveal distinct meta-measure characteristics

**Fig 3:**
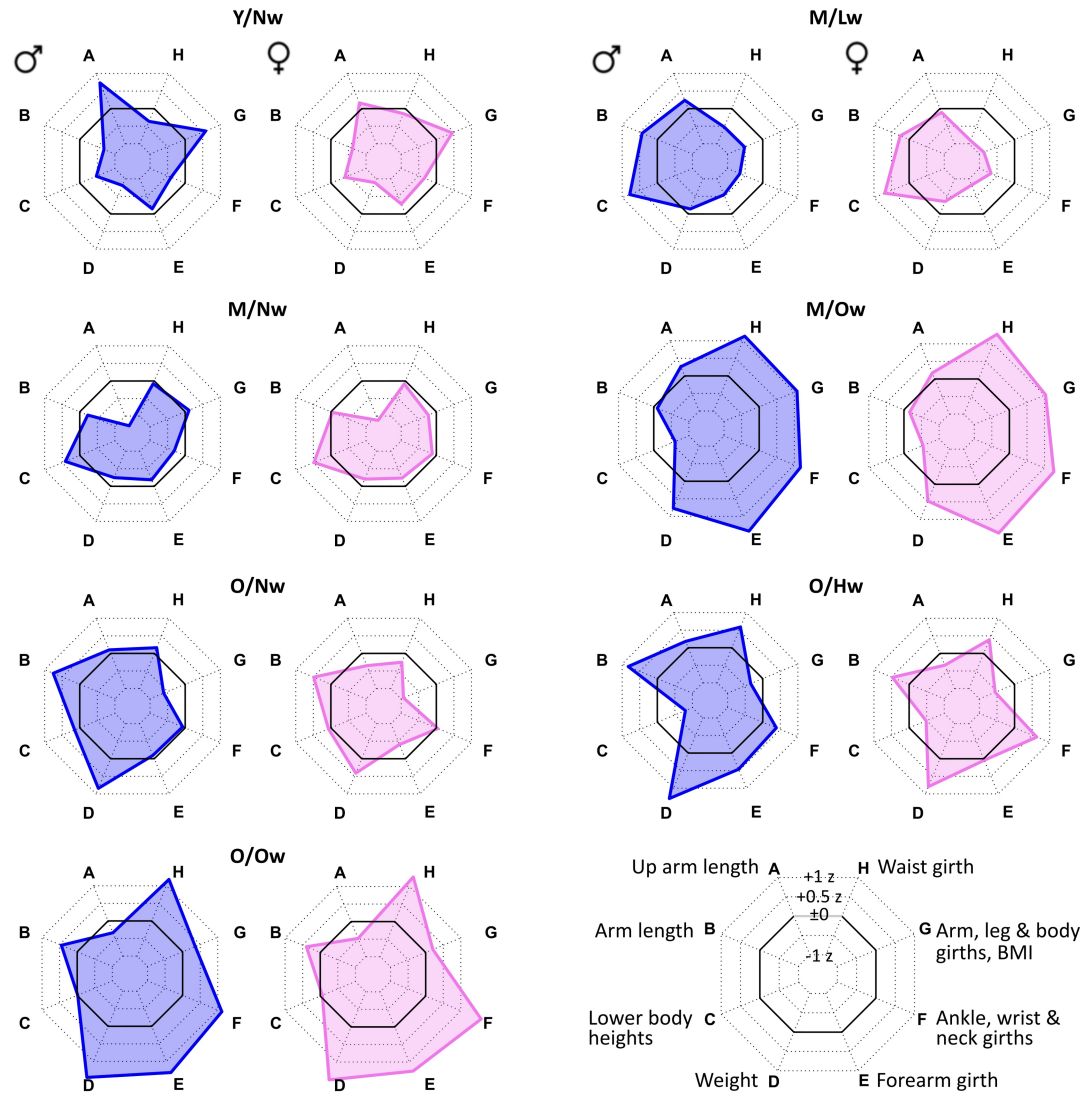
Body types identified in the LIFE Child study are characterized by specific body shapes. The bodygrams visualize the meta-measures of the body types as polar diagrams. Coordinates are given in standard deviation (Z-) units (see legend).

The body types were shown to associate with specific age and BMI characteristics. However, they were defined using all meta-measures which enables characterization of the respective whole body shapes. To relate body types to the corresponding body shapes, we visualize the values of the eight meta-measures using a polar diagram representation called ‘bodygram’ (Fig 3). The black polygon refers to Z = 0 and thus to the mean value of each meta-measure averaged over the sample. In the following the terms ‘big’, ‘small’, ‘long’ and ‘short’ relate to these average values [13].

In general, bodygrams for each body type are almost similar for boys and girls. The degree of agreement between the male and female bodygrams is largest for overweight body types, which reflects the lack of gender specifics for overweight participants. Detailed inspection of the bodygrams reveals also gender specific details, such as (relatively) short upper arms in girls compared to boys in ‘Y/Nw’-, ‘O/Nw’- and ‘O/Hw’-types. Meta-measures ‘B’ (arm length) and ‘D’ (weight) increase in older body types according to their age courses, as discussed above. Meta-measures ‘E’ and ‘H’, referring mainly to girth measures, show very large values in the overweight body types ‘M/Ow’ and ‘O/Ow’. These two body types, however, differ in length measures, especially in arm and lower body lengths (meta-measures ‘A’, ‘B’ and ‘C’). Meta-measure ‘F’, which includes the body measures ankle, wrist and neck girths, reveals an even more complex pattern. Young participants (‘Y/Nw’) and older overweight body types (‘M/Ow’ & ‘O/Ow’) associate with large values. Contrarily, small values of ‘F’ are exclusively observed in the lower and normal weight body types ‘M/Lw’ and ‘O/Nw’.

Hence, the bodygrams show that the body types are related to distinct body shape characteristics. In general, larger areas encompassed by the bodygrams associate with bigger body shapes, while smaller areas refer to thinner and/or longer bodies.

### Body typing changes with age

**Fig 4:**
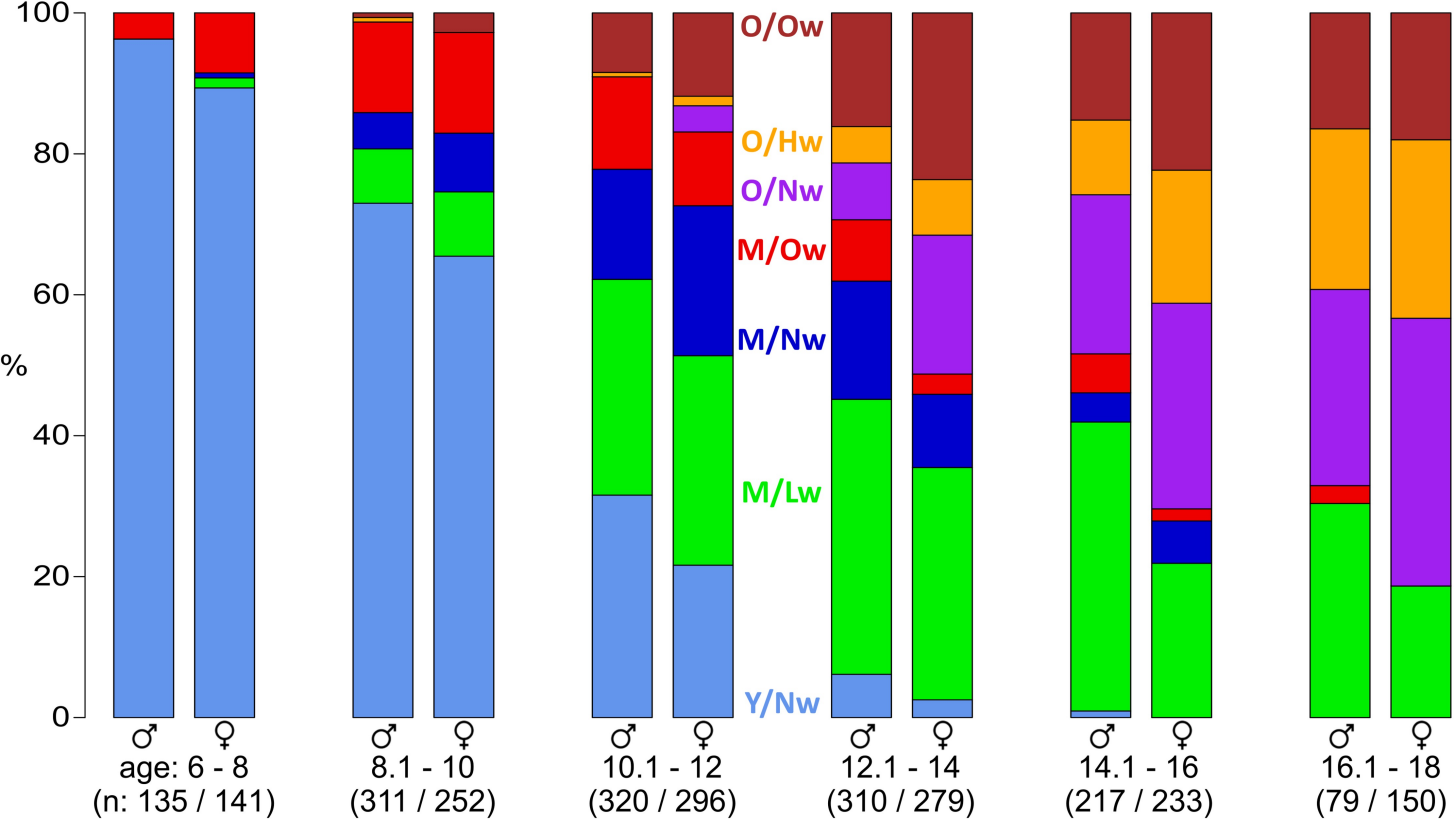
Body type distributions change upon development. The distribution of body types stratified for different age ranges shows a systematic shift form ‘Y’ via ‘M’ to ‘O’-types.

We found seven body types of distinct body shapes which associate with specific age ranges as supported by the age-dependent frequency distribution of the body types (Fig 4): As expected, ‘younger’, ‘medium age’ and ‘older’ body types emerge and outgrow in successive order. Interestingly, the amount of older-age body types is higher in girls compared to boys of the same nominal age. For example, for 12- to 18-years-old participants, 32% of girls, but only 18% of boys, are assigned to the body types ‘O/Nw’, ‘O/Hw’ and ‘O/Ow’. This effect might be explained by the earlier maturation of female bodies [22], which is reflected in an earlier shift into ‘M’- and ‘O’-body types for girls, respectively. Moreover, these results show that the body types itself are relatively sex- and age-invariant. They include approximately the same number of participants from both sexes and from different ages within the specific age ranges of the body types.

## Summary and conclusion

We analyzed 3D-body scanning data collected in the LIFE Child study with 2,700 participants covering an age range between 5 and 18 years. Our analysis provides a set of eight meta-measures representing major dimensions of body shapes. These meta-measures reflect changes of the body proportions from childlike to adolescent. Upon development we found a general shift to body shapes with–in relation to body height–larger girths and longer arms, paralleled by an over-proportional increase of weight. Bimodal courses of meta-measures related to lower body lengths and to upper arm length, respectively, indicate that their development proceed on different time scales.

Clustering of the meta-measures of all participants provides seven body types. These body types differentiate between normal weight and overweight participants and thus they further stratify BMI-based classes: We found three body types with high BMI which associate with specific body shapes and age ranges. Age-dependent BMI-curves of the body types are shifted with respect to age-course of the mean BMI in the German population reference. In this respect, body typing based in 3D-body scanning provides a multidimensional approach to anthropometrically characterize population cohorts in detail. It complements simple one-dimensional indices such as BMI or waist-to-hip ratio. Such anthropometrical characterization of developing and aging populations constitutes a novel option to investigate onset and progression of obesity and other civilization diseases in children.

## Supporting information

S1 Text Supplementary text. (PDF)

S1 Table Body scanner data table. It contains sex, age, body type, and height- and Z-normalized body measures of all 2,735 participants.
(XLSX)

S2 Table Table of body measures and meta-measure assignment. (XLSX)
