# Supplementary material for: Body typing of children and adolescents using 3D-body scanning
Source: PLoS One. 2017 Oct 20;12(10):e0186881. doi: 10.1371/journal.pone.0186881 (PMC5650166; doi:10.1371/journal.pone.0186881)
Supplement: S1 Text — (PDF) [file pone.0186881.s001.pdf]

Body typing of children and adolescents using 3D-body scanning

Henry Loeffler-Wirth*, Mandy Vogel, Toralf Kirsten, Fabian Glock, Tanja Poulain, Antje Körner, Markus Loeffler, Wieland Kiess¶, Hans Binder¶

Supplementary text

1. Clustering of body measures into eight meta-measures 2
2. Clustering of LIFE Child participants into seven body types 3
3. Description of LIFE Child body types 5
4. Age-dependent BMI-trajectories of the body types 6
5. Body types represent clusters of consistent BMI SDS level 7
6. References 8

1. Clustering of body measures into eight meta-measures

A feature map of preprocessed body scanner data is generated using SOM machine learning (1). A SOM size of 50 x 50 was used to achieve stable clustering of the 46 body scanner measures (see (2) for details). The body measures arrange in a two-dimensional grid after SOM training such that measures with similar profiles across the sample locate at the same or at close positions, whereas measures with dissimilar profiles are located in distant regions of the map. Figure S 1a shows the localization of the body measures within the feature map. Each dot marks at least one individual measure. If multiple measures are located at the same position, their number is indicated by the color scale given within the figure. Blue dots represent positions with one single measure, dark red dots represent positions with six measures. Then the feature map was segmented into clusters as described in (13). This way we obtained eight clusters of anthropometric measures termed meta-measures, each containing between 1 and 15 individual body measures. Meta-measures are annotated by capital letters ‘A’ to ‘H’ as illustrated in the main article. The corresponding cluster regions are highlighted in Figure S 1a. Meta-measures along the left edge of the feature map refer mainly to girth measures, whereas those in the right part of the map mainly refer to length measures. The complete list of assignments of body measures to meta-measures is given as S2 Table. The localization of length and girth measures in opposite areas of the map reflects anti-correlated profiles, i.e. larger girths on average associate with smaller lengths and vice versa. Please remind that data was adjusted for body height during preprocessing. The meta-measures consequently refer to scaled body measures in relation to body height.

Figure S 1: Clustering of body measures using self-organizing map (SOM). Meta-measures are defined by clusters of single body measures in the feature map and annotated by capital letters ‘A’ to ‘H’. Positions of the single measures are indicated by the dots. The color represents the number of single measures mapped to each position (see scale bar). Gray areas indicate the cluster regions. They are annotated by most prominent measures and the number of measures within the clusters.

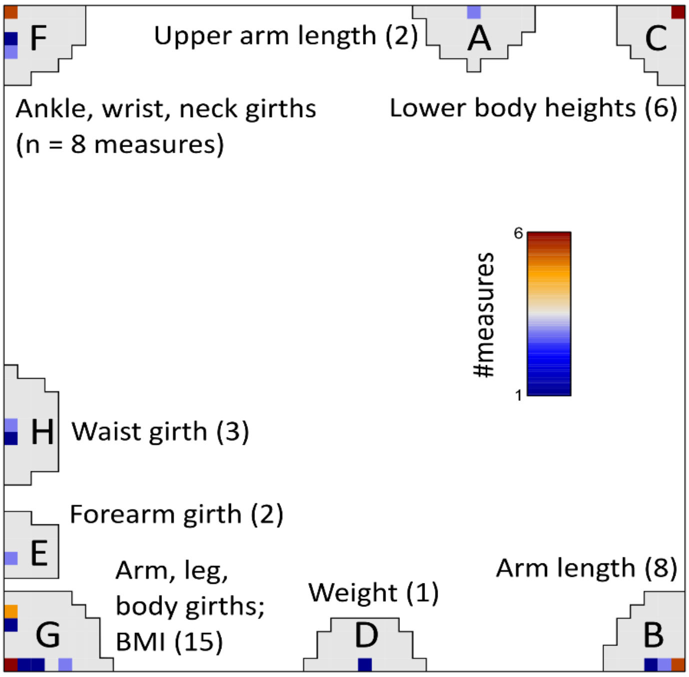

2. Clustering of LIFE Child participants into seven body types

The eight meta-measures characterize the participants’ body shape with reduced dimensionality compared to the 46 original body measures. We used them for diversity analysis of all 2,735 participants in terms of consensus clustering with 100-fold bootstrapping. The resulting consensus matrix was hierarchically clustered and reveals several clusters which group as blue quadrat-like areas along the diagonal in Figure S 2a. Dynamic tree cutting (3) of the corresponding cluster dendrogram then provided seven body types.

Stability of body type clusters was estimated by comparison of intra-cluster consensus and inter-cluster consensus (2), representing the mean connectivity of all feature pairs of the same and of two differing clusters, respectively. The former measure estimates the stability of the individual clusters, the latter one estimates the degree of overlap between two clusters (Figure S 2b and c). All body types show intra-cluster consensus ≥ 0.70 with an average value of 0.75, indicating compact and stable clustering. Body types of the adult population of Leipzig (LIFE ADULT cohort) show markedly smaller values of intra-cluster consensus (≥ 0.44, on average

0.62 (2)), which means that body typing in children is more stable than in adults as seen by comparison of body-type clustering of LIFE Child and LIFE Adult.

Inter-cluster consensus estimates the degree of overlap between two body types. These values are used as distance measures to generate a dendrogram of body types using hierarchical clustering with single linkage (Figure S 2c). It reveals a successive, linear clustering of the body types on a very low level of mutual overlap (average inter-cluster consensus of 0.05) rather than forming distinct branches. These results consequently indicate that the defined body types are distinct and non-overlapping.

Figure S 2: Body types detected in LIFE Child form distinct and robust clusters. (a) Consensus cluster map of the participants. Light to deep blue coloring indicates the increasing frequency of pairwise appearance of different participants in the same clusters as determined in 100-fold bootstrapped clustering. Body types are defined by separate branches of the dendrogram shown above the heatmap. (b) Intra-cluster consensus values for the body type clusters reflect the degree of stability. (c) Hierarchical cluster dendrogram of body type clusters. Inter-cluster consensus values were used as similarity measure.

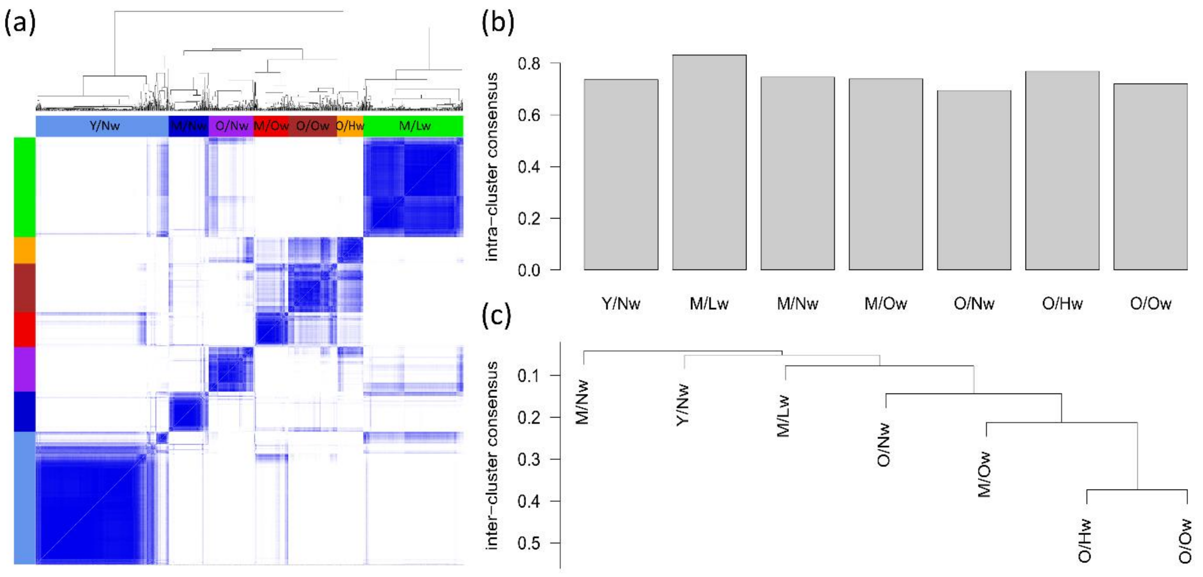

3. Description of LIFE Child body types

Table S 1: Description of the body types stratified according to male and female participants.

Body type	# Individuals [1]		Age (y) [2]	BMI [2]	BMI SDS [2]	Weight (kg) [2]	Height (cm) [2]	WTH [2]
Y/Nw	852	♂ 486	9.0 ± 1.7	16.6 ± 2.2	-0.37 ± 0.72	30.7 ± 6.9	135.3 ± 9.1	0.84 ± 0.04
		♀ 366	8.7 ± 1.5	16.2 ± 1.8	-0.40 ± 0.70	29.2 ± 5.7	133.4 ± 8.4	0.82 ± 0.05
M/Lw	640	♂ 356	13.0 ± 2.0	17.7 ± 2.1	-0.76 ± 0.81	46.9 ± 11.6	161.2 ± 12.8	0.80 ± 0.05
		♀ 284	12.7 ± 2.2	16.9 ± 1.8	-1.02 ± 0.72	42.6 ± 8.8	157.9 ± 10.7	0.76 ± 0.05
M/Nw	255	♂ 127	11.8 ± 1.5	18.2 ± 2.4	-0.38 ± 0.59	42.4 ± 9.2	151.7 ± 8.9	0.82 ± 0.05
		♀ 128	11.5 ± 1.6	18.5 ± 2.3	-0.29 ± 0.64	42.9 ± 8.6	151.6 ± 8.7	0.79 ± 0.05
M/Ow	220	♂ 128	11.1 ± 2.2	24.7 ± 5.8	0.91 ± 0.67	56.5 ± 18.3	150.1 ± 10.3	0.87 ± 0.07
		♀ 92	10.0 ± 2.0	23.8 ± 4.5	1.08 ± 0.70	50.2 ± 13.9	144.1 ± 9.0	0.87 ± 0.06
O/Nw	287	♂ 96	15.0 ± 1.4	21.5 ± 2.6	-0.11 ± 0.60	66.7 ± 10.4	175.6 ± 7.3	0.78 ± 0.06
		♀ 191	14.8 ± 1.8	20.4 ± 1.9	-0.26 ± 0.58	56.3 ± 7.4	165.9 ± 5.8	0.73 ± 0.05
O/Hw	169	♂ 61	14.8 ± 1.9	24.0 ± 4.0	0.33 ± 0.73	74.1 ± 16.2	175.3 ± 8.7	0.79 ± 0.08
		♀ 108	15.1 ± 1.6	22.8 ± 2.4	0.21 ± 0.48	61.6 ± 9.3	164.0 ± 6.2	0.74 ± 0.05
O/Ow	312	♂ 125	13.4 ± 1.9	26.4 ± 4.6	0.90 ± 0.60	72.6 ± 16.9	165.2 ± 8.7	0.86 ± 0.07
		♀ 187	13.6 ± 2.0	27.4 ± 5.5	0.98 ± 0.51	71.3 ± 18.1	180.8 ± 7.1	0.81 ± 0.07

1: total number of participants in the body type, and numbers of male and female subgroups

2: average value ± standard deviation

4. Age-dependent BMI-trajectories of the body types

Body types collect participants from broad age ranges, which potentially cover different stages of development. We evaluated relation of BMI and age in the cross-sectional sample using running averages of the BMI separately for each body type. This approach smooths the relatively large scatter of BMI values of the individuals of each body type (Figure S 3). Note that our body types integrate multidimensional information on whole body proportions, which goes beyond the simple, one-dimensional BMI index. For a rough comparison we interpret trends of the body type related BMI curves compared to the 50%-percentile (P50) reference line of German children and adolescents (4):

The curves of 'Y'- and 'M'-types proceed virtually parallel to the P50 line indicating that growth velocity (BMI increment per year) of these body types agrees with average growth velocity in the reference population where boys show a larger variability of the curves than girls. Note that BMI-curves of age related body types are partly overlapping for boys (especially 'M/Ow' and 'O/Ow', and also 'Y/Nw', 'M/Nw' and 'O/Nw'), whereas the curves for girls show a more sequentially order ('M/Ow' and 'O/Ow') or are simply shifted in the BMI level ('Nw'-types).

Figure S 3: BMI characteristics change with age at time of measurement and reveal specific ranges of the individual body types. The BMI-values were smoothed for each body type using running average of a two-year sliding window. The gray curves indicate 50 percentile of German children and adolescents. Each dot represents an individual measurement.

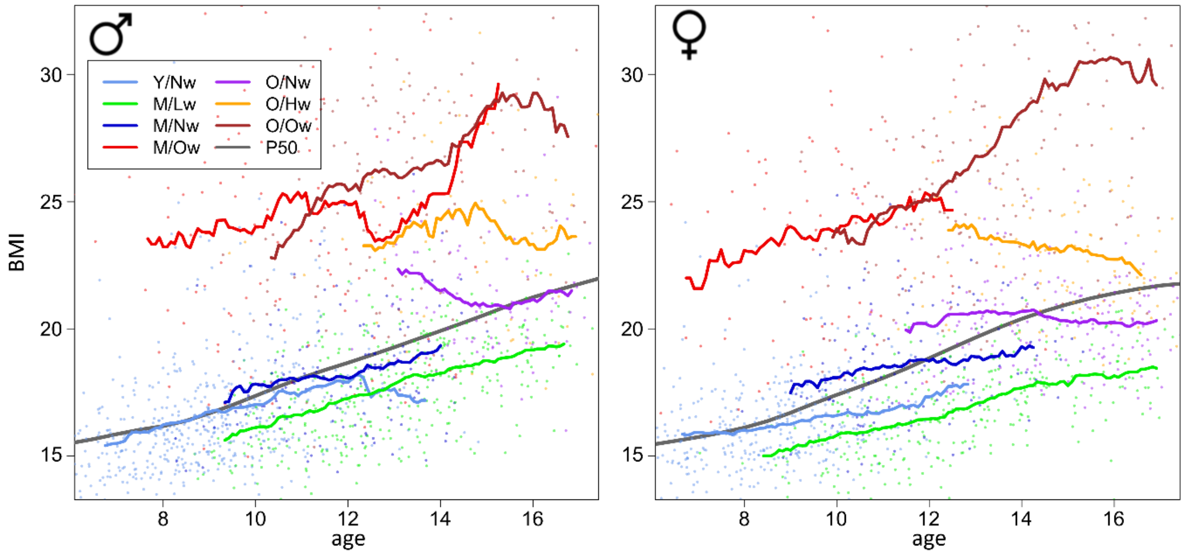

5. Body types represent clusters of consistent BMI SDS level

Typically developmental curves are normalized with respect to the population mean and standard deviation (SDS scores). Age-dependent BMI-SDS curves are very similar compared to the original BMI curves of the body types (compare Figure S 4a and Figure 5 in main manuscript). In particular, overlap of age ranges and deviations from the population reference are virtually identical for BMI and BMI-SDS.

Scatterplots of body height and weight were generated and reveal curves shifted in parallel to the reference (Figure S 4b). Distances between the body type isolines and the reference in this plot almost perfectly correlate to average BMI SDS values of the body types (r=0.99 in male, r=0.94 in female participants). This indicates that age-specific characteristics as represented by SDS values are conserved in our body typing.

Figure S 4: BMI SDS characteristics change with age at time of measurement. (a) BMI SDS of participants smoothed for each body type using running average and a two-year window. (b) Body height and weight of participants smoothed using running average. The numbers indicate minimum, median and maximum age in the body types. The gray curves indicate 50 percentile of German children and adolescents. Body types are colored according to legend given in (a).

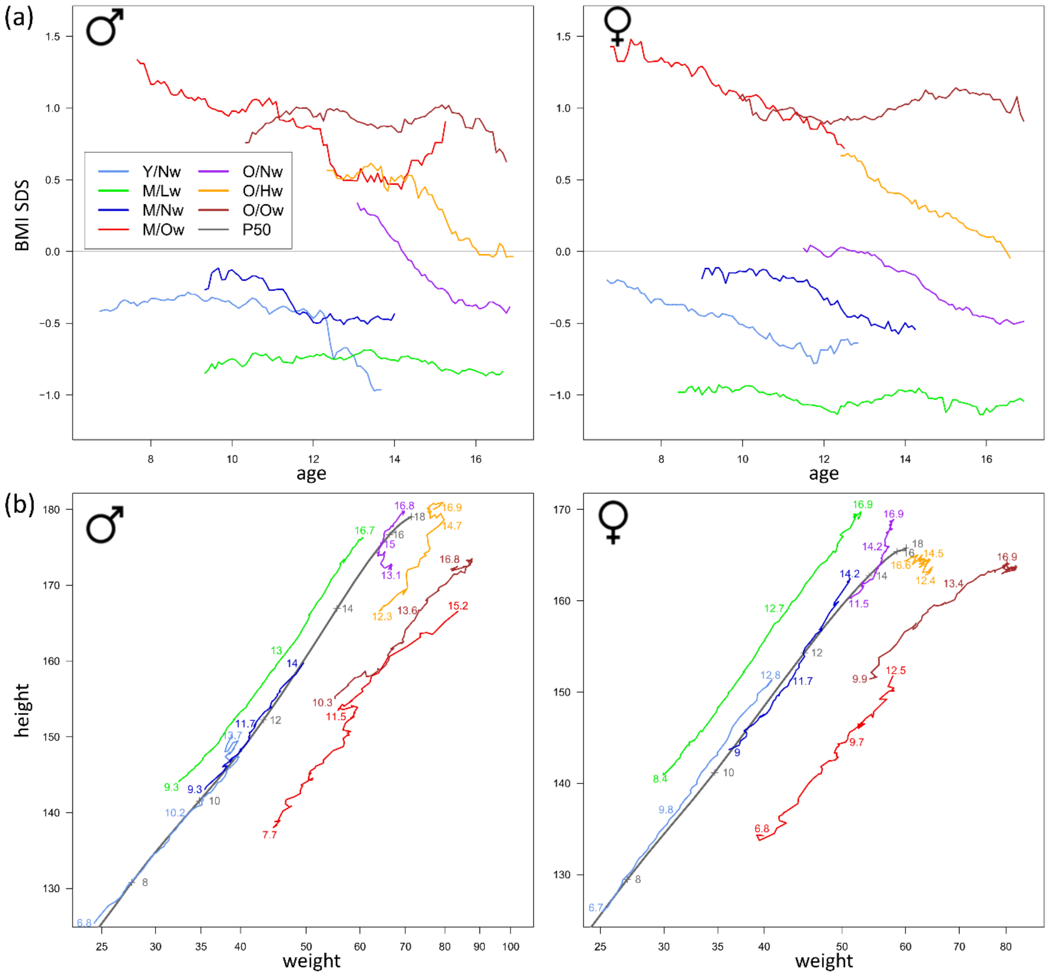

6. References

1. Kohonen T. The self-organizing map. Proc IEEE [Internet]. 1990 [cited 2010 Sep 13]; Available from: http://ieeexplore.ieee.org/xpls/abs_all.jsp?arnumber=58325

2. Löffler-Wirth H, Willscher E, Ahnert P, Wirkner K, Engel C, Loeffler M, et al. Novel Anthropometry Based on 3D-Bodyscans Applied to a Large Population Based Cohort. PLoS One [Internet]. 2016 [cited 2016 Jul 29];11(7):e0159887. Available from: http://www.ncbi.nlm.nih.gov/pubmed/27467550

3. Langfelder P, Zhang B, Horvath S. Defining clusters from a hierarchical cluster tree: the Dynamic Tree Cut package for R. Bioinformatics [Internet]. 2008 Mar 1 [cited 2015 Apr 22];24(5):719–20. Available from: http://www.ncbi.nlm.nih.gov/pubmed/18024473

4. Robert Koch-Institut. Referenzperzentile für anthropometrische Maßzahlen und Blutdruck aus der Studie zur Gesundheit von Kindern und Jugendlichen in Deutschland (KiGGS) [Internet]. Berlin; 2013. Available from: http://www.rki.de/DE/Content/Gesundheitsmonitoring/Gesundheitsberichterstattung/GesundAZ/Content/K/Koerpermaße/Körpermaße.html
